# Exosomes on Endometrial Cancer: A Biomarkers Treasure Trove?

**DOI:** 10.3390/cancers14071733

**Published:** 2022-03-29

**Authors:** Alexandros G. Sykaras, Konstantinos Christofidis, Ekaterini Politi, Stamatios Theocharis

**Affiliations:** 1 First Department of Pathology, Medical School, National and Kapodistrian University of Athens, 11527 Athens, Greece; alexander.sykaras@gmail.com (A.G.S.); konstantinos.christofidis@gmail.com (K.C.); 2 Department of Cytopathology, Aretaieion Hospital, Medical School, National and Kapodistrian University of Athens, 11528 Athens, Greece; ekpoliti@med.uoa.gr

**Keywords:** endometrial cancer, extracellular vesicles, exosomes, liquid biopsy, biomarkers, microRNA

## Abstract

**Simple Summary:**

Endometrial cancer (EC) is one of the main causes of cancer-related death among women. Despite the advances in gynecologic oncology, the incidence of EC is on the rise, and the relative 5-year survival remains unchanged in the last decade. This creates an urgent need for a deeper understanding of the pathogenesis of EC that will result in new diagnostic and therapeutic approaches. Ideally, we need biomarkers of clinical value that can be analyzed in non-invasive ways. Exosomes are abundant in biological fluids and are considered as a valuable source of biomarkers. Exosomes are extracellular vesicles that originate from cells and function as shuttles, transferring biomolecules between cells. These biomolecules have a critical role in the cancer pathogenesis and progression and in regulating the tumor cells’ proliferation and metastasis, and they represent attractive therapeutic targets. Here, we review the functions of exosomes in EC, focusing on the potential biomarkers of diagnostic and prognostic significance or potential therapeutic use.

**Abstract:**

Endometrial cancer (EC) is one of the main causes of cancer-related death among women. In the last decade, the incidence of EC is on the rise, and the relative 5-year survival remains unchanged. This creates a dire need for new diagnostic and therapeutic approaches that can only result from a deeper understanding of the pathogenesis of the disease. In this direction, exosomes are under heavy research, with two main aims: to identify the potential diagnostic and prognostic markers and to develop technologies based on their use as therapeutic vectors targeting EC cells. Exosomes are widely available in all bodily fluids and are sources of ideal biomarkers for liquid biopsies. They are extracellular vesicles containing DNA, RNA, lipids, and proteins, which they transfer between cells, serving multiple functions and being implicated in both the physiological processes and the pathogenesis of diseases. Of all the biomolecules contained in exosomes, microRNAs (miRNAs) seem to have the most clinical utility in the diagnosis and treatment of EC. Exosomal miRNAs mediate the communication between EC cells, cancer-associated fibroblasts (CAFs), and tumor-associated macrophages (TAMs) and have a pivotal role in the tumor cells’ proliferation, epithelial to mesenchymal transition (EMT), and the formation of a tumor microenvironment. They participate in many processes that are tied to carcinogenesis and cancer progression, and they are therefore considered as attractive therapeutic targets. Here, we review the functions of exosomes in EC, focusing on potential biomarkers of diagnostic and prognostic significance or potential therapeutic use.

## 1. Introduction

### 1.1. Endometrial Cancer

Endometrial cancer (EC) is the second most common malignancy of the female genital tract, and the sixth most common female cancer, with much higher incidence rates in western countries in comparison with African or Asian countries, according to Global Cancer Statistics 2020 [1]. Several risk factors have been associated with sporadic EC, namely obesity, diabetes, polycystic ovary syndrome (PCOS), infertility, and the use of tamoxifen, whereas inherited EC develops as a manifestation of Lynch and Cowden syndrome [2]. EC is mainly a disease of postmenopausal women, with a mean age of onset at 65 years. Only a small minority of cases occur in premenopausal women and very rarely before the age of 40 [3]. In the last decade, the incidence of EC has been growing significantly, and the mean age of onset has been dropping in all western countries, mainly due to obesity, diabetes, parity, the reduced number of early-life hysterectomies and the increased life expectancy [4]. On the other hand, the relative 5-year survival has not improved in many years [5]. In patients with localized EC, the 5-year overall survival (OS) is excellent (95%), whereas patients with metastatic disease have poor outcome (5-year OS less than 20%) [6].

The diagnosis of EC is based on morphology, and several histological subtypes of EC are included in the latest World Health Organization (WHO) classification of the tumors of the uterine corpus [7]. The most common histologic subtype of EC is endometrioid EC (EEC), which is histologically graded as low-grade (grade 1–2, which constitutes the majority of EEC cases) or high-grade (grade 3). In contrast, tumors with a non-endometrioid histology, namely serous, clear cell, dedifferentiated–undifferentiated, mixed, mucinous gastric-type, and squamous cell carcinomas, neuroendocrine carcinomas, and uterine carcinosarcomas, as well as mesonephric and mesonephric-like adenocarcinomas, are not graded but considered as high-grade tumors with aggressive biological behavior [8]. Nevertheless, histologically similar ECs, particularly the high-grade tumors, may follow a different clinical course. Moreover, the substantial interobserver variability in the diagnosis of high-grade EC subtypes makes the stratification of EC patients problematic [9].

A molecular subcategorization of EC based on The Cancer Genome Atlas-Uterine Corpus Endometrial Cancer (TCGA-UCEC) datasets was attempted to ameliorate the diagnostic objectivity and prognostic relevance of EC classification [10]. These molecular subtypes were validated in subsequent studies, and surrogate immunohistochemical markers have been identified for the classification of EC specimens into these subtypes in the routine clinical practice [11,12,13]. Thus, EC is now divided into four molecular subtypes; DNA Polymerase E (POLE)-ultramutated; MMR (MisMatch Repair)-deficient/MSI (microsatellite instability)-hypermutated; p53-mutant/copy number high-serous like; and NSMP (non-specific molecular profile)/copy number low-endometrioid groups [14]. Patients with EC of the POLE-ultramutated and NSMP subtypes usually have a more favorable prognosis. MMR-deficient patients have an intermediate prognosis, whereas p53-mutant EC is characterized by the worst prognosis. The most recent guidelines for the EC patients’ stratification integrate the molecular profile with other clinicopathologic parameters, such as tumor histological type, grade, and differentiation, as well as (International Federation of Gynecology and Obstetrics) FIGO stage, depth of myometrial invasion, and lymphovascular space invasion (LVSI) [15]. Thus, six risk categories have been introduced (low, intermediate, high-intermediate, high, advanced, metastatic) in an attempt to treat EC patients properly [15,16]. The current molecular–histological classification of EC is complex and has limitations; therefore, there is an urgent need for the identification of biomarkers that could be incorporated in the prognostic stratification algorithms

The standard treatment of EC is surgical, namely a total hysterectomy, bilateral salpingo-oophorectomy, in combination with peritoneal effusion cytopathological examination cytology, for an adequate surgical and pathological staging. Moreover, in reproductive-age patients, fertility-sparing surgery must be considered when possible [17]. The most recent guidelines propose that EC in low-risk patients should be treated with surgery without adjuvant therapy [18]. Adjuvant brachytherapy should be considered for intermediate risk patients, whereas external beam radiation therapy (EBRT) and chemotherapy are additional options for high/high-intermediate risk patients. For patients with advanced or metastatic EC, surgery should only be used in cases where it is possible to obtain a complete cytoreduction with no residual disease. When this is not possible, a systemic palliative radiotherapy treatment should be considered together with other options, such as chemotherapy and hormonotherapy [19].

Unfortunately, to date, there does not exist a non-invasive screening method that can detect pre-malignant, dysplastic lesions and intraepithelial carcinomas of the endometrium in order to achieve an early-stage diagnosis and make appropriate treatment plans. It is obvious that sensitive, specific, and peripherally available biomarkers are urgently needed. Moreover, endometrial pathology is characterized by marked heterogeneity. Therefore, a screening method that would be able to monitor the individual cancer status of a patient could open the way to personalized medicine [20]. In this context, liquid biopsy samples can be used for genomic and proteomic analyses that will reveal new biomarkers showing all the necessary traits to serve as diagnostic, prognostic, and/or therapeutic tools.

### 1.2. Liquid Biopsy

A liquid biopsy, also known as fluid biopsy or fluid phase biopsy, is the sampling and analysis of non-solid biological tissue for tumoral elements, namely circulating tumor cells and cell-free tumor DNA (ctDNA), as well as extracellular vesicles (EVs), cell-free microRNAs (cfmiRNAs), mRNA, long noncoding RNAs (lncRNAs), small RNA, circulating cell-free proteins, and tumor-educated platelets (TEPs) [21]. Many body fluids can be used for liquid biopsy, including blood, urine, uterine aspirates, saliva, cerebrospinal fluid, and pleural effusions. Ideally, a patient should be receiving treatment in accordance with the molecular profile of the treated neoplasm. Unfortunately, the neoplastic molecular profile might change in the course of the cancer’s progression. Moreover, even within a single neoplasm, there might exist significant heterogeneity. Lastly, not all tumors and their metastases are easily accessible by needle biopsy, incommoding sampling. Thus, it is often difficult, if not unattainable, to treat a tumor according to its specific molecular signature at each specific moment. Liquid biopsy offers a solution to this by its facile access to the tumor cells or tumoral products of all the subclones at any specific time point. In this way, serial liquid biopsies can better track the spatial and temporal heterogeneity of tumors than the analyses of neoplastic tissue samples, leading to a better monitoring of the disease’s evolution and opening the possibility of the development of better treatment strategies [22,23].

Uterine aspirates are particularly useful liquid biopsy samples for the identification of biomarkers related to EC. These samples can be obtained by minimally invasive techniques, and their study sheds light on the profile of proteins and EVs secreted in the endometrial fluid [24]. Moreover, they provide valuable information about the mutational profile and the intra-tumoral heterogeneity of EC [25], even pre-operatively and in tumors that are not classified histologically [26]. Although the exosomal profile of uterine aspirates has been analyzed deeply in physiological processes (embryo implantation) and endometriosis, it remains largely unexplored in EC [27].

### 1.3. Exosomes

Extracellular Vesicles (EVs) represent a heterogeneous group of membrane-bound lipid particles that originate from the plasma membrane or the endosomal system and are secreted from cells. EVs, released from both healthy and cancer cells, are abundant in body fluids and mediate cell-to-cell communication by shuttling DNA, RNA, lipids, metabolites, and proteins [28]. In this way, EVs are implicated in numerous physiological processes but also participate in the formation of the tumor microenvironment (TME) and the cancer’s progression. Given that the EV cargo is representative of the parental cell physiology–pathology, EVs are currently considered as treasure troves for the discovery of biomarkers, with diagnostic, prognostic, and therapeutic value in cancer and other diseases, such as renal pathology [29,30,31].

Based on their size and mechanism of production, extracellular vesicles are divided into two categories, ectosomes and exosomes, both containing DNA, RNA, lipids, metabolites, and proteins. Apoptotic bodies are also considered as, EVs but they are released from dying cells and have a distinct content (organelles, chromatin) and a clearly different proteomic profile to ectosomes or exosomes. Ectosomes bud outward off the surface of the cellular plasma membrane. They are divided into microvesicles (MVs), microparticles (MPs), as well as large vesicles with a diameter of ~50 nm to 1 mm. Exosomes have an endosomal origin and a diameter of ~40 to 160 nm [32]. Their formation starts with an invagination of the plasma membrane and the endocytosis of extracellular constituents and fluid, creating the early sorting endosomes (ESEs) that turn into late sorting endosomes (LSEs). After a second invagination of the LSE membrane, intraluminal vesicles (ILVs) are generated. The latter contain multivesicular bodies (MVBs) that can fuse with the plasma membrane, releasing exosomes [33].

Exosomes can be markedly heterogeneous, due to differences in the cells of origin, size, content, and function. They are thought to play a role in intercellular communication, as well as in maintaining the cellular homeostasis. This can act positively or negatively in the development of many diseases, including cancer [34]. Exosomes can easily be sampled by liquid biopsy as they are abundant in all body fluids. Cutting-edge cancer research focuses on finding the specific exosome (most notably miRNA) profiles of different cancers, as well as of the different stages of specific cancers. Thus, exosomes could serve as both diagnostic and prognostic biomarkers. Moreover, disease progression could be monitored by the easily accessible liquid biopsy-derived exosomes [35]. Specific biomolecules contained in the exosomes are part of many cellular pathways, some of which are linked to cancer. As a result, selective inhibition or amplification of these biomolecules could have therapeutic effects. Moreover, exosomes can be turned into carriers of biomolecules and drugs that they can deliver to specific recipient cells, avoiding many barriers of the immune system that would stop an artificial carrier [36]. There is already a lot of research and many promising results in the use of exosomes for diagnostic, prognostic, and therapeutic purposes in many cancers, including EC [37].

miRNAs are the most studied biomolecules contained in exosomes. They are small, non-coding RNAs that have been shown to have tumorigenic, as well as anti-oncogenic potential. Normally, they take part in cellular development, proliferation, differentiation, senescence, and apoptosis [38]. A specific gene can be targeted by many miRNAs, and a specific miRNA can target more than 200 genes. Twenty to thirty percent of genes have been shown to be miRNA targetable [39].

In order to properly study the EVs obtained by liquid biopsy, they must first be isolated. It is crucial that the isolation method is of high yield and purity and does not damage the EVs, as this would tamper with their function as biomarkers or therapeutic vectors. Several methods have been developed for the isolation of exosomes, including density-based ultracentrifugation, precipitation, and immunoaffinity capture-based and microfluidics-based techniques [40]. The different methods available have different advantages and disadvantages, and an ideal method, being fast, simple, inexpensive, non-damaging to the EVs and with a high yield and purity, does not exist [41]. As far as endometrial cancer is concerned, many EV isolation methods have been used, the most important of which are ultracentrifugation, ultra-filtration, polymer-based precipitation with size-based purification, and polymer-based precipitation. The latter has proven to be the best method as it does not damage the EVs, demonstrates a high EV yield, and allows easy subsequent RNA profiling. This method can process many samples simultaneously, while being simple and easy to use. Unfortunately, it is also relatively expensive [42].

## 2. Exosomes in the Pathophysiology of Endometrial Cancer

**Figure 1:**
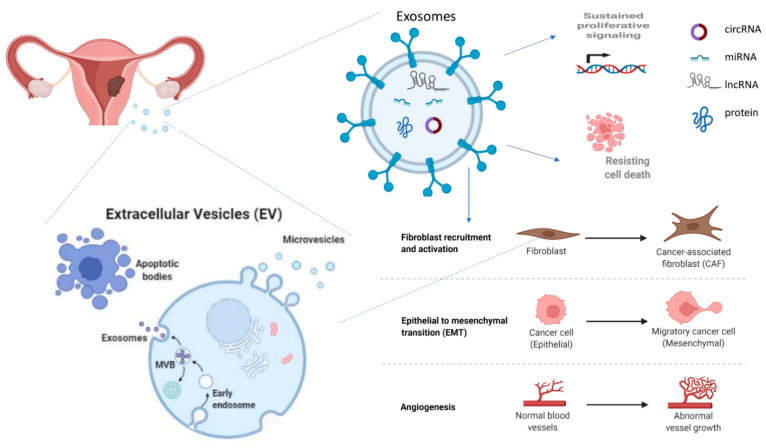
The role of exosomes in the progression of endometrial cancer. Exosomes are released by EC cells as well as cancer-associated fibroblasts and tumor-associated macrophages. They contain molecules (proteins, miRNAs, circRNAs, and lncRNAs) that participate in cancer progression by enhancing tumor proliferation, inhibiting apoptosis, inducing angiogenesis and epithelial to mesenchymal transition. miRNA: microRNA; circRNA: circular RNA; EC: endometrial cancer.

The characterization of exosomes in gynecological cancers, in particular EC, is rather limited in comparison with other solid tumors [27]. However, there is a great ongoing effort to explore the role of exosomes in the pathogenesis of EC as there is already ample evidence that exosomes are connected with EC’s angiogenesis and epithelial-mesenchymal transition (EMT), as well as the survival, growth, and invasive and metastatic potential of EC cells (Figure 1) [37]. Exosomes participating in the pathophysiology of EC cancer are released from EC cells as well as Cancer-Associated Fibroblasts (CAFs) and Tumor-Associated Macrophages (TAMs) and have a pivotal role in the communication between these cell populations.

### 2.1. Exosome-Mediated Interaction between EC Cells and CAFs

Maida et al. used the Ishikawa EC cell line as an experimental model and demonstrated that EC cell-derived exosomes transfer miRNAs to endometrial fibroblasts, altering the miRNA expression profile of the recipient fibroblasts. This finding indicates that exosomes participate in the communication between EC cells and CAFs in the human endometrium [43]. LncRNA nuclear enriched abundant transcript 1 (NEAT1) is upregulated in exosomes released from EC CAFs. Exosomal NEAT1 is transferred to EC cells where it downregulates the expression of miR-26a/b-5p. miR-26a/b-5p suppresses the expression of Chitinase 3 like 1 (YKL-40/CHI3L1), a driving force of tumor growth and metastasis, via targeting STAT3. Exosomal NEAT1 is also able to promote tumor growth in a xenograft model of HEC-1A EC cells. More specifically, the administration of CAFs that overexpress NEAT1 leads to enhanced EC tumor volume and increased proliferation capacity, as demonstrated by the elevated percentage of proliferation index marker Ki-67-immunopositive tumor cells. The effect of NEAT1 was mediated via the regulation of the miR-26a/b-5p-STAT3-YKL-40 axis [44].

**Figure 2:**
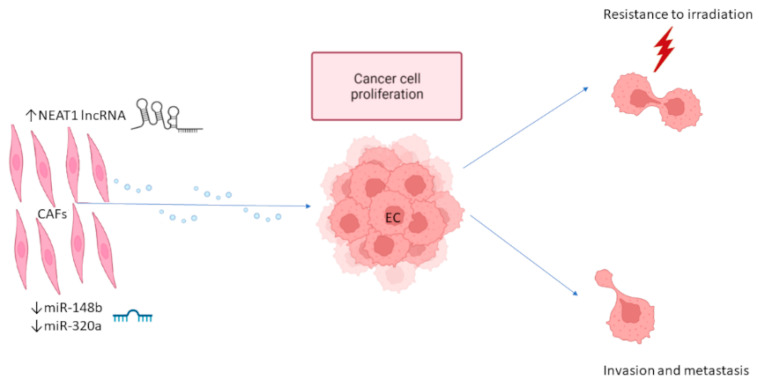
CAF-derived exosomes promote endometrial cancer growth. Exosomes derived from EC CAFs are enriched in lncRNA NEAT1 that has pro-oncogenic activity and is depleted in tumor suppressor miRNAs miR-148b and miR-320a. These CAF-derived exosomes are up-taken by EC cells, promoting EC cell proliferation, migration, and invasion, as well as resistance to irradiation. CAFs: cancer-associated fibroblasts; miRNA: microRNA; EC: endometrial cancer.

EC CAF-derived exosomes may contain downregulated miRNAs with tumor suppressor functions. Li et al. demonstrated that EC CAFs contain and secrete in exosomes markedly lower levels of miR-148b compared to normal fibroblasts. miR-148b gets transferred to the EC cells, where it suppresses DNA Methyltransferase 1 (DNMT1) that, if left unsuppressed, promotes EMT and EC progression. Therefore, the downregulation of miR-148b in EC CAF-derived exosomes is partly responsible for EC cell invasion and metastasis. Thus, the overexpression of miR-148b might serve as a therapeutic method in EC [45]. Similarly, the expression of miR-320a is downregulated in EC CAFs, EC CAF-derived exosomes, and EC cells. miR-320a inhibits the proliferation of EC cells by suppressing the HIF1α/VEGFA axis [46]. Both HIF1a and VEGFA are considered as poor prognostic markers for EC [47,48]. The downregulation of HIF1α/VEGFA levels is also linked to an increased radiosensitivity of EC cells [49], and exosomes overexpressing miR-320a may be used as a therapeutic agent in EC patients (Figure 2) [46].

The communication between EC cells and endometrial stromal cells is critical for EC cancer progression. FOXL2 was identified as one of the four Differentially Expressed Genes (DEGs) between EC and normal tissue samples [50]. FOXL2 is a tumor-suppressor gene whose expression is significantly downregulated in EC cell lines and EC samples. Low expression of FOXL2 in EC correlates significantly with poor prognosis. FOXL2 is targeted by miR-133a, amiRNA that is upregulated in EC samples and EC-derived exosomes that can be taken up by normal stromal cells. In this way, exosomal miR-133a mediates the communication between EC cells and endometrial stromal cells, facilitating the progression of EC through the involvement of the adjacent stroma [50].

### 2.2. Exosome-Mediated Communication between EC Cells and Tumor Infiltrating Immune Cells

More macrophages are present in EC tissue than in benign endometrium. Most of the tumor-associated macrophages (TAMs) that infiltrate EC are polarized M2 macrophages. TAM infiltration of EC tissue positively correlates with cancer progression as an increased number of macrophages is significantly associated with high grade, advanced stage, lymph node involvement and LVSI [51,52].

In EC, hypoxia also seems to be associated with a higher tumor grade, lymph node metastasis, and tumor chemoresistance. It has been shown that, under hypoxia conditions, EC cells produce exosomes that have immunomodulatory effects. An EC KEL cell line cultured under hypoxic conditions secretes a significantly higher number of exosomes than normoxic KEL cells. These exosomes showed a marked increase in miR-21 expression and promoted M2-like polarization of the macrophages when added to a culture of the THP-1 monocyte cell line, an effect attenuated by miR-21 inhibitors. These data suggest that hypoxia may facilitate the formation of the necessary immune microenvironment for EC development by inducing the transfer of exosomal miR-21 from cancer cells to neighboring monocytes [53]. miR-21 expression is also upregulated in the hypoxic EFE-184 EC cell line and in serum samples from EC patients [54,55]. The overexpression of miRNA-21 augments the proliferation and migration of EC cells via the downregulation of PTEN [56,57]. Moreover, the expression of miR-21-3p correlates with the decreased OS of EC patients [58].

**Figure 3:**
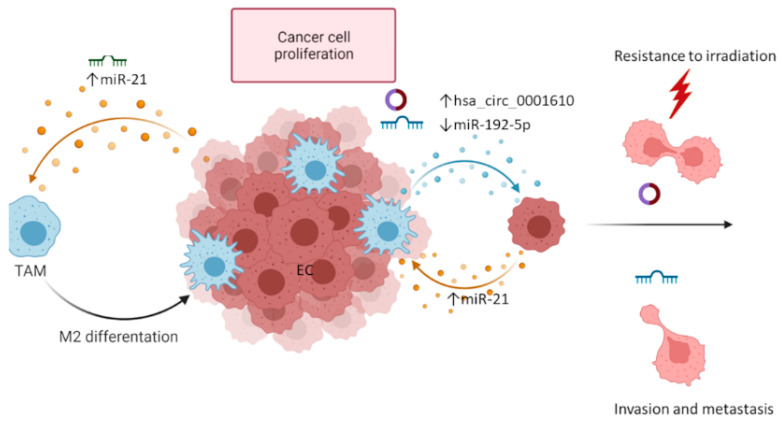
Exosomes mediate communication between endometrial cancer cells and tumor-activating macrophages to sustain cancer progression. EC cells release exosomes enriched in miR-21, promoting the differentiation of the neighboring monocytes to M2 macrophages. Polarized M2 macrophages release exosomes enriched in circRNA hsa_circ_0001610 that are absorbed by EC cells, promoting tumor proliferation and resistance to irradiation. Additionally, TAM-derived exosomes transferred to EC cells are depleted in miR-192-5p, a miRNA with tumor suppressor activity, resulting in an uncontrolled proliferation of the EC cells and epithelial to mesenchymal transition. miRNA: microRNA; circRNA: circular RNA; EC: endometrial cancer.

EC M2-polarized TAMs release exosomes that increase the viability and the proliferation of EC cells and attenuate their radiosensitivity. This effect is mediated by the overexpression of exosomal circRNA hsa_circ_0001610. hsa_circ_0001610 negatively regulates the expression of miR-139-5p and increases the expression of cyclin B1, a target of miR-139-5p, that promotes radioresistance. The administration of exosomes in a xenograft EC model caused a post-irradiation increase in tumor growth and promoted EMT. These effects were augmented with the administration of exosomes that overexpressed hsa_circ_0001610, whereas the silencing of hsa_circ_0001610 abolished the ability of the delivered exosomes to weaken the radiosensitivity of EC cells [59]. Another recent study provided additional evidence demonstrating that TAMs promote the proliferation of EC cells and EMT via exosome transfer. TAM-derived exosomes express low levels of miR-192-5p, a miRNA that suppresses the IRAK1/NF-κB pathway, promoting EC progression. These data point to a novel therapeutic target and raise the possibility that overexpression of miR-192-5p can be considered as a therapeutic approach for EC (Figure 3) [60].

Immune system cells are tied to carcinogenesis in multiple ways, the exosomes they release being one of them. M2 macrophages, through their secreted cytokines, can induce the epigenetic upregulation of estrogen receptor alpha (ERα) expression, which enhances the proliferative effects of estradiol in EC cells and increases the endometrium’s sensitivity to estrogens, thus promoting type I (estrogen-dependent) EC carcinogenesis [61]. Estrogen was shown to have a tumorigenic effect on EC in an estrogen receptor beta (ΕRβ)-dependent way, by downregulating miR-765 and upregulating PLP2. The overexpression of PLP2 leads to an enhanced proliferation and EMT via activation of the Notch pathway. This is rivaled by the antitumoral effect of the CD45RO-CD8+ tumor-infiltrating T cells’ exosomal miR-765. Recently, exosomes released by CD8+ T cells have been under the spotlight in the field of cancer immunotherapy. In EC, the findings of Zhou et al. highlight a potential therapeutic approach based on CD8+ T cell-derived exosomes with a high expression of miR-765 for EC patients with overactivation of the ERβ/miR-765/PLP2/Notch signaling axis [62].

### 2.3. EC-Derived Exosomes Involved in Cancer Progression

LncRNAs are considered to be involved in EC tumorigenesis and progression. Exosomal lncRNA Deleted Lymphocytic Leukemia 1 (DLEU1) has been shown to promote the proliferation, migration, and invasive ability of EC cells by regulating the miR-381-3p/E2F3 axis in vitro. Exosomal DLEU1 is consequently up-taken by EC cells where it downregulates MiR-381-3p, causing upregulation of E2F3, the target gene of miR-381-3p. As a result, the knockdown of DLEU1 and/or miR-381-3p and E2F3 downregulation disrupts this critical axis for EC’s progression and may be used as a therapeutic tool [63].

Additionally, exosomes are implicated in the progression of EC in women with polycystic ovary syndrome (PCOS). Serum exosomes derived from PCOS patients promoted the EC’s ability to invade and migrate when added to Ishikawa and HEC-1 EC cell lines. In order to find the differentially expressed miRNAs between PCOS patients and normal control patients, a sequence-based analysis of exosomal miRNA and a real-time PCR were carried out and found miR-590-3p and miR-27a-5p to be upregulated—the second markedly so—and miR-375a-3p and miR-19b-3p to be downregulated. Exosomal miR-27a-5p directly targets SMAD4 and downregulates SMAD4 mRNA and protein expression, promoting the migration and invasion of EC cells [64].

## 3. Exosomal Biomarkers in Biological Fluids of EC Patients

The analysis of biological fluids obtained from EC patients has provided valuable information about exosomal biomarkers with a diagnostic or prognostic value. Most of these studies have been performed in cohorts of EC patients with mixed EC subtypes or cohorts with an unknown histological-molecular EC subtypes’ profile. Table 1 summarizes the exosomal biomarkers identified by the study of EC cell lines and the profiling of exosomes present in liquid biopsies of EC patients.

### 3.1. Plasma-Serum Biomarkers

#### 3.1.1. Proteins

Proteomic analysis of plasma exosomes from EC patients revealed that they are enriched in lectin galactoside-binding soluble 3 binding protein (LGALS3BP) protein. LGALS3BP promotes EC growth and human umbilical vein endothelial cells (HUVECs) angiogenesis in vitro and in vivo via the PI3K/AKT/VEGFA signaling pathway. LGALS3BP was found to be progressively elevated in patients with atypical endometrial hyperplasia and low-grade and high-grade EC, correlating with a poor prognosis. Therefore, plasma exosome LGALS3BP may represent a biomarker of diagnostic and prognostic value [65]. Additionally, Annexin 2 (ANXA2) protein is enriched in plasma EVs isolated from EC patients. ANXA2 has been suggested as a diagnostic EC biomarker given that its expression has the accuracy and the sensitivity to distinguish EC patients. Moreover, ANXA2 levels correlate with non-endometrioid histological type, high grade histology, advanced FIGO stage, and high risk of recurrence. Therefore, plasma EV ANXA2 is considered as a promising biomarker of diagnostic and prognostic value [66].

#### 3.1.2. miRNAs

A comparative analysis of the plasma-derived exosomal miRNA profile in EC patients and healthy subjects revealed that miR-15a-5p, miR-106b-5p, and miR-107 are significantly upregulated in the plasma exosomes of EC patients. Importantly, miR-15a-5p serves as a potentially excellent diagnostic biomarker of early EC, allowing the discrimination between stage I EC and healthy controls. Moreover, the miR-15a-5 expression correlates with clinicopathologic parameters (tumor size, muscular layer infiltration, and positive p53 staining) but not with a histological subtype with similar expression levels in EC with endometrioid or non-endometrioid histology [67]. Another study with a similar aim identified a panel of miRNAs (miR-142-3p, miR-146a-5p, and miR-151a-5p) that are significantly upregulated in the plasma of EC patients compared to healthy controls. This 3-miRNA signature has been suggested as a promising diagnostic biomarker. From these miRNAs, only miR-151a-5p is upregulated in the plasma exosomes of EC patients [68]. Additionally, serum exosomal miR-93 and miR-205 expression has been reported to have prognostic value in EC. miR-93 is significantly upregulated in the serum of EC patients and its expression correlates with smoking, high tumor grade, advanced FIGO stage, metastatic spread, and reduced OS. In contrast, the expression of miR-205 is significantly downregulated and is inversely associated with smoking, lymph node metastasis, advanced FIGO stage, and decreased OS [69].

#### 3.1.3. circRNAs

Circular RNAs (circRNAs) are another class of non-coding RNAs. Although originally thought not to be worth extensive research, they have recently drawn a lot of attention as they have been linked to carcinogenesis. It was found that circRNAs can act as miRNA competitive inhibitors, tampering with the latter’s ability to bind to their targets. Moreover, circRNAs have been shown to be enriched in exosomes. A comparative analysis of the exosomal circRNA profile from EC patients and healthy control sera showed 275 differentially expressed circRNAs, the majority being upregulated. Most of the differentially expressed circRNAs were linked to pathways associated with neoplastic migration and invasion, namely the focal adhesion pathway, the ECM-receptor interaction pathway and the regulation of actin cytoskeleton pathway. hsa_circ_0109046 and hsa_circ_0002577 were shown to be the most important differentially expressed circRNAs and can prove to be useful diagnostic biomarkers [70].

### 3.2. Peritoneal Lavage Exosomal Biomarkers

Roman-Canal et al. examined the miRNA profile of EVs isolated from peritoneal lavage to identify biomarkers for EC. Of the 114 miRNAs that were differentially expressed between EC patients and healthy controls, eight (miRNA-383-5p, miRNA-10b-5p, miRNA-34c-3p, miRNA-449b-5p, miRNA-34c-5p, miRNA-200b-3p, miRNA-2110, and miRNA-34b-3p, all significantly downregulated in EC) demonstrated a predictive value [71]. Many of these miRNAs are related to tumor progression. In particular, miRNA-10b and miRNA-34b are downregulated in serous EC tissues, and the low expression of miRNA-10b is significantly associated with decreased OS [72]. On the contrary, miRNA-200b acts as an inhibitor of EMT and metastatic spread by targeting Zinc Finger E-Box Binding Homeobox (ZEB) ZEB1 and ZEB2 but is surprisingly upregulated in serous EC tissues [73]. Although the source of EVs present in the peritoneal lavage is not confirmed (EC cancer cells or reactive mesothelial cells?), it is possible that the spread of EVs to the peritoneal cavity mediates metastasis [71].

### 3.3. Urine Exosomal Biomarkers

Srivastava et al. studied the expression of miRNAs in urine-derived exosomes from patients with EC and symptomatic patients without a diagnosed EC and found a panel of 57 differentially expressed miRNAs, with hsa-miR-200c-3p being enriched the most [74]. miR-200c acts as a tumor suppressor that inhibits tumor growth and blocks EMT by targeting ZEB1 and ZEB2 [75]. Notably, the expression of miR-200c is also dysregulated in other malignancies, such as ovarian, breast, and pancreatic cancer. Therefore miR-200c cannot be solely used as a specific biomarker of EC [38]. In contrast, other studies of the cell-free urinary miRNA content found differentially expressed miRNAs of diagnostic value only in the supernatant but not in the exosomal fraction [76,77].

### 3.4. Uterine Blood–Uterine Aspirate Biomarkers

Blood and urine are the most common sources of liquid biopsies for research and clinical purposes. However, proximal bodily fluids could also be a good source of EC biomarkers. For EC, this was put to the test by the analysis of the expression levels of 52 proteins in liquid biopsies of uterine fluid in trying to identify proteomic signatures that differentiate between EC and control patients and between the different subtypes of EC. Twenty-eight proteins were markedly upregulated in EC patients, from which the combined use of metalloproteinase 9 (MMP9) and pyruvate kinase (KPYM) as diagnostic biomarkers showed 94% sensitivity and 87% specificity for EC. Importantly, the use of this two-protein diagnostic panel allowed the differentiation between EC and benign conditions that cause thickening of the endometrium in both premenopausal and postmenopausal women. Furthermore, a diagnostic panel of β-catenin (CTNB1), exportin-2 (XPO2), and macrophage-capping protein (CAPG) showed 95% sensitivity and 96% specificity for differentiating between the two main EC subtypes, endometrioid and serous [25,78]. Although the exosomal profile of uterine blood has not been studied yet, a comparative study examined the diagnostic and prognostic utility of endothelial and monocytic microparticles (MPs), a subtype of EVs measuring 0.1–1 μm in size, isolated from the peripheral and uterine blood of EC patients [79]. More MPs were detected in patients with EC than in the healthy controls, but there was no significant correlation between MP levels and histologic grade or FIGO clinical stage. Specifically, the MP count in uterine blood was higher than the count in peripheral blood. This finding raises the possibility that exosome markers may be detected earlier in uterine than in peripheral blood, and a detailed characterization of the exosomal profile in uterine blood may be needed [79].

**Table 1:** Exosomal biomarkers in endometrial cancer.

Origin	Biomarker	Biomarker Type	Expression	Function/Significance	Potential Application	Ref
EC CAFs	NEAT1	lncRNA	↑	EC growth	Therapy	[44]
EC CAFs	miR-148b	miRNA	↓	EC cell invasion, EMT	Therapy	[45]
EC CAFs	miR-320a	miRNA	↓	EC cell proliferation	Therapy	[46]
EC TAMs	hsa_circ_0001610	circRNA	↑	EC cell proliferation Resistance to irradiation	Therapy	[59]
EC TAMs	miR-192-5p	miRNA	↓	EC cell proliferation EMT	Therapy	[60]
CD45RO-CD8+ TILs	miR-765	miRNA	↑	EC growth inhibition	Therapy	[62]
EC cells	miR-133a	miRNA	↑	Communication between EC and stromal cells	Therapy	[50]
EC cells	DLEU1	lncRNA	↑	EC cell proliferation, migration and invasion	Therapy	[63]
EC cells (hypoxia)	miR-21	miRNA	↑	EC cell proliferation and migration, ↓OS **	Prognosis Therapy	[53,56,57,58]
hUMSCs	miR-503-3p	miRNA	↑	EC growth inhibition	Therapy	[80]
Serum from stage III adenocarcinoma patients	hsa_circ_0109046 hsa_circ_0002577	circRNAs	↑	EC migration and invasion	Diagnosis	[70]
Serum *	miR-93	miRNA	↑	Correlation with smoking, tumor grade, advanced stage, metastasis, ↓OS	Diagnosis Prognosis	[69]
Serum *	miR-205	miRNA	↓	Correlation with smoking, lymph node spread, advanced stage, ↓OS	Diagnosis Prognosis	[69]
Plasma	LGALS3BP	Protein	↑	EC growth, angiogenesis	Diagnosis Prognosis	[65]
Plasma	miR-15a-5p	miRNAs	↑	Correlation with tumor size and depth of invasion, similar expression in EEC and non-EEC patients	Diagnosis of early EC	[67]
Plasma	miR-106b-5p miR-107	miRNAs	↑	Unknown, similar expression in EEC and non-EEC patients	Diagnosis	[67]
Plasma *	miR-151a-5p	miRNA	↑	Unknown	Diagnosis	[68]
Plasma from EEC (*n* = 19) and non-EEC (*n* = 3) patients.	miR-10b-5p, miR- 34b-3p, miR-34c- 5p, miR-34c-3p, miR-449b-5p, miR-200b-3p, miR-383-5p, miR-2110	miRNAs	↓	EC progression ↓ miR-10b-5p correlates with ↓OS miR-200b-3p inhibits EMT	Diagnosis	[71]
Plasma from EEC (*n* = 26), non-EEC (*n* = 12) and other EC subtypes (*n* = 3) patients.	ANXA2	Protein	↑	Correlates with high grade, non-endometrioid subtype, advanced stage, and increased risk of recurrence	Diagnosis Prognosis	[66]
Urine	hsa-miR-200c-3p	miRNA	↑	EC growth inhibition EMT block	Diagnosis	[74]
Serum from PCOS patients	miR-590-3p miR-27a-5p	miRNAs	↑	miR-27a-5p promotes EC cell migration-invasion	Diagnosis	[64]
Serum from PCOS patients	miR-375a-3p miR-19b-3p	miRNAs	↓	Unknown	Diagnosis	[64]

EC: Endometrial Cancer; EEC: Endometrioid Endometrial Cancer; PCOS: Polycystic ovary syndrome; CAFs: Cancer-associated fibroblasts; miRNA: micro RNA; circRNA: Circular RNA; EMT: epithelial to mesenchymal transition; TAMs: tumor-associated macrophages; OS: overall survival * EC subtype is not mentioned in the manuscript, but the authors used a 3-tier grading system for the histological classification, indicating that EEC cases are included in this cohort. ** Analysis performed in a cohort of 124 patients from the TCGA-Uterine Corpus Endometrial Cancer database.

## 4. Exosomes as Therapeutic Agents

The discovery of exosomal miRNAs that play a crucial part in EC pathogenesis, as well as their target genes, can play a critical role in creating new therapeutic approaches against EC. Engineered exosomes that overexpress tumor-suppressor miRNAs may become valuable weapons in the fight against EC cancer progression. The analysis of the EC miRNA profile provides information about downregulated miRNAs and reveals novel therapeutic targets.

The restitution of miRNAs downregulated in EC by miRNA-loaded exosomes represents an interesting option of target therapy. Analysis of the EC miRNA profile provides information about the targets of this approach. For example, it was recently demonstrated that miR-499a-5p is markedly downregulated in EC tissue and cell lines. miR-499a-5p inhibits EC proliferation both in vitro and in vivo by targeting VAV3 and suppresses EC growth, angiogenesis, and metastasis [81]. The enhancement of miRNA-499a-5p expression may be considered as an additional therapeutic approach in EC.

Human umbilical cord mesenchymal stem cell (hUCMSC)-derived exosomes are considered as ideal tools for target-based therapies. Engineered exosomes that overexpress tumor-suppressor miRNAs can be delivered by hUCMSCs to EC cells and inhibit their proliferation. Using this approach, Li et al. targeted EC cells with miR-302a overexpressing exosomes and impaired their proliferation and migration by suppressing the cyclin D1 levels and inactivating the AKT signaling pathway [82]. Furthermore, the overexpression of miR-503-3p in hUCMSC-derived exosomes suppressed the expression of mesoderm-specific transcript (MEST) in EC cells and inhibited tumor growth [80].

## 5. Conclusions

EC, being one of the most common cancers of women, must be urgently analyzed in depth if we are to hope to understand its pathogenesis and discover new diagnostic and prognostic biomarkers, as well as therapeutic tools. Exosomes have a pivotal role in the pathogenesis of EC, orchestrating the communication between the tumor cells and the tumor microenvironment. In parallel, the exosomes in the biological fluids of EC patients contain molecules that can be used as biomarkers of diagnostic and prognostic value. Although the study of EC exosomes is a hot research topic, technical limitations have impeded its progress and important aspects remain to be answered. An outstanding question is whether the different histological and molecular EC subtypes, which have different prognoses, are characterized by distinct exosomal profiles and subtype-specific exosomal biomarkers. Until now, we have not identified any EC subtype-specific exosomal biomarkers that could potentially facilitate the development of precise and targeted treatments. Exosomes are treasure troves in our quest for reliable EC biomarkers, and their detailed characterization is a prerequisite for achieving this aim.
